# Post-silking Phosphorus Recycling and Carbon Partitioning in Maize Under Low to High Phosphorus Inputs and Their Effects on Grain Yield

**DOI:** 10.3389/fpls.2019.00784

**Published:** 2019-06-12

**Authors:** Chao Wang, Peng Ning

**Affiliations:** ^1^College of Natural Resources and Environment, Northwest A&F University, Yangling, China; ^2^Department of Plant Nutrition, College of Resources and Environmental Sciences, China Agricultural University, Beijing, China; ^3^State Key Laboratory of Plant Genomics, Institute of Genetics and Developmental Biology, Chinese Academy of Sciences, Beijing, China

**Keywords:** phosphorus, carbohydrate, grain yield, phosphorus use efficiency, *Zea mays*

## Abstract

Phosphorus (P) recycling and carbon partitioning are crucial determinants of P-use efficiency and grain yield in maize (*Zea mays*), while a full understanding of how differences in P availability/plant P status affect these two processes underlying yield formation remains elusive. Field experiments were conducted for 3 years to investigate the maize growth, P remobilization, and carbohydrate accumulation in leaves and developing ears of plants receiving low to high P inputs. In plots that 75 kg P_2_O_5_ ha^-1^ and above was applied (corresponding to 7.5 mg kg^-1^ and higher Olsen-P concentration in 0–20 cm soil layer), no additional response occurred in leaf area, ear growth, and grain yield. Despite the higher P uptake with P fertilization above this threshold, the lack of additional plant growth and yield resulted in decreased P-use efficiency. Regardless of P application rates, P remobilization to the ear during the first half of the grain filling phase preferentially came from the stem (50–76%) rather than from leaves (30–44%), and with a greater proportion in the inorganic P (Pi) form over organic P fractions. Leaf photosynthesis was maintained under P-limiting conditions due to the greater P investment in organic P pools than Pi. More and larger starch granules were found in the bundle sheath cells at silking or 21 days after silking (DAS) than under P-sufficient conditions. The amount of total carbohydrate production and export was lower in the P-deficient plants than the high-P plants, corresponding to decreased leaf size and lifespan. Nonetheless, similar or significantly greater starch levels were observed in both cob and kernels at silking and 21 DAS, implying there was an adequate carbohydrate supply to the developing ears under the diminished kernel sink of the P starvation. In addition, there was a strong correlation between the accumulation rates of carbon and remobilized P in the developing kernels, as well as between carbon and total P. Overall, the results indicated that diminished sink size and lower capacity of carbon deposition may limit yield formation in P-deficient maize, which in turn imposes a feedback regulation on reducing carbon and P remobilization from source leaves. An integrated framework considering post-silking P recycling and carbon partitioning in maize and their effects on grain yield has been proposed.

## Introduction

Phosphorus (P) is one of the most important macronutrients for plants, and its deficiency usually causes reduced crop growth and lower grain yield (Fredeen et al., 1989; Plénet et al., 2000a; Assuero et al., 2004). For achieving higher yield, farmers prefer to apply high rates of P fertilizer to cropland, e.g., in China often twice as much fertilizer P and nitrogen (N) is applied than is recovered in crops (Chen et al., 2011). It is estimated that the average soil-available P concentration in China cropland has increased from 7.4 mg kg^-1^ in 1980 to 20.7 mg kg^-1^ in 2006 following the high rates of P fertilizer application (Li et al., 2011). This excessive P use in turn adversely affects environmental quality and human well-being (Sharpley et al., 1996; Vitousek et al., 2009). Despite differing viewpoints on the state of global P reserves, these sources are rapidly being depleted which poses a serious threat to global food security (Cordell et al., 2009). Therefore, the questions raised here include how to use P efficiently under P-limiting conditions and whether high P inputs could further improve P uptake and increase maize grain yield or alter the pattern of P recycling and plant dry matter production.

Besides P-acquisition from field soil, the internal P recycling in plants (e.g., remobilization) is a crucial direction to improve P-use efficiency of crops and relieve stress to P reserves and alleviate environmental issues from excess P fertilizer application (Childers et al., 2011; Veneklaas et al., 2012). During the grain filling period P stored in vegetative organs is progressively recycled to support the growth of developing grains, especially under P deficiency (Marschner et al., 1996). The senescing leaves could export at least 50% of P, and often much more (Aerts, 1996; Ning et al., 2013), while adequate concentrations of P are important to maintain high rates of photosynthesis (Rychter and Rao, 2005). Jones (1983) revealed that critical P concentrations in shoots required for maximum aerial biomass change from 5.5 mg g^-1^ before silking to 2.3 mg g^-1^ at maturity on a dry weight (DW) basis. The lowest inorganic P (Pi) concentration for maximal photosynthesis was 0.6 mmol m^-2^ in maize leaves (Usuda and Shimogawara, 1991). From this point of view, large amounts of P export from leaves would weaken the carbon (C) capture, while the retention of leaf P may reduce P-use efficiency. It is critical to investigate the responses of post-silking P recycling to P availability in order to better understand its influences on the C capture and grain yield, as well as the P-use efficiency.

Improving internal P use efficiency of maize also needs to result in higher grain yield which highly depends on post-silking C production and allocation to developing ears (Lee and Tollenaar, 2007; Ning et al., 2013). In cereals, a decrease of vegetative P concentration often occurs after anthesis or flowering (Veneklaas et al., 2012), while most of the grain dry matter is obtained by photosynthesis during this period (Cliquet et al., 1990; Tollenaar et al., 2004; Lee and Tollenaar, 2007). Previous studies revealed that lower P availability affects not only maize growth (especially leaf growth), but also photosynthesis, C fixation and export, and partitioning (Rao and Terry, 1989; Usuda and Shimogawara, 1991; Plénet et al., 2000b). Nonetheless, there is little research in maize to determine post-silking redistribution of P for optimal C gain across a range of soil-P supplies.

In addition, high grain-P content is not necessarily desirable for humans from a nutritional perspective and should be reduced (Veneklaas et al., 2012). A survey across a number of grain crops (cereals, legumes, and oil seeds), including maize, found that the harvest index (ratio of grain yield to whole-plant dry matter at maturity) is generally lower than the phosphorus harvest index (ratio of grain P to whole-plant P content) (Veneklaas et al., 2012), implying a faster movement of P than carbohydrates to developing grains. On the other hand, it seems that P movement and sugar transport from leaves to grains are closely linked, and the former is largely driven by C demands in sinks, but not by P requirement (Marshall and Wardlaw, 1973; Veneklaas et al., 2012), while high Pi concentrations in grains would suppress starch formation (Smidansky et al., 2002, 2003). Therefore, it is necessary to understand how the P remobilization and C production and transport from maize source leaves to ear are coordinated during the grain filling.

In the present work, field experiments were conducted using a range of low to high P-application rates for 3 years. Phosphorus uptake, remobilization, and carbohydrate accumulation in source leaves and grains were investigated in maize after silking. The objectives were to assess the impacts of different P inputs on the pattern of P uptake and recycling during maize grain filling, C production and movements to developing ears, and C and P relationships and association with final grain yield.

## Materials and Methods

### Site Description and Experimental Design

Field experiments were conducted in 2014-2016 at the China Agricultural University Shangzhuang Experimental Station (40°8′20″N, 116°10′47″E), Beijing, China. The soil is a calcareous alluvial with silt loam texture (FAO), a typical type in the North China Plain. The monthly mean temperature during maize growing seasons in May, June, July, August, and September was 22, 25, 28, 26, and 20°C in 2014, 22, 25, 27, 26, and 21°C in 2015, and 22, 26, 29, 27, and 22°C in 2016, respectively. The amount of rainfall from May to September was 33, 79, 116, 42, and 105 mm in 2014, 42, 57, 140, 60, and 142 mm in 2015, and 47, 56, 306, 29, and 41 in 2016, respectively. In addition, 20, 40, and 14 mm of water was irrigated on June 20th, July 8th and July 18th in 2014, respectively.

The experiments were nested in a long-term study begun in 2009 which includes six treatments of P-fertilizer inputs, i.e., 0, 50, 75, 100, 150, and 300 kg P_2_O_5_ ha^-1^ (referred to as P0, P50, P75, P100, P150, and P300, respectively). Each treatment was fixed in the same plots every year. The present study was carried out in the 2014 to 2016 growing seasons. The chemical properties of the 0–20 cm (topsoil) and 20–40 cm (subsoil) soil layers of each treatment before sowing are listed in Supplementary Table S1. Soil pH was determined with a glass electrode pH meter (soil:water = 1:1, w:v). Soil organic matter was determined using a titration method after oxidation with K_2_Cr_2_O_7_ (Schollenberger, 1927). Soil mineral N (NO_3_^-^ and NH_4_^+^), Olsen-P, and NH_4_OAc extractable K were determined as previously described (Ning et al., 2015). Cation exchange capacity was determined as described by Hajek et al. (1972). The six treatments were arranged in a randomized complete block design with four replications. Each plot was 5 m wide and 7 m long (35 m^2^). Maize hybrid “Zhengdan 958” was selected for the study and was sown on 6 May each year in rows 0.6 m apart to achieve a stand density of 66,000 plants ha^-1^. 1 day before sowing, all the P fertilizer (as calcium superphosphate), 75 kg N ha^-1^ (as urea), and 80 kg K_2_O ha^-1^ (as potassium sulfate) were broadcast-applied and incorporated into the top 0–15 cm soil layer by rotary tillage. An additional 150 kg N ha^-1^ was later top-dressed, split equally between the V8 (8th fully expended leaf) and silking stages. Additional top dressing of 40 kg K_2_O ha^-1^ was applied at silking. Weeds were controlled by pre-emergent herbicide application followed by manual hoeing as needed.

### Plant Sampling

Whole-plant samples were harvested from all six treatments (from P0 to P300) at silking, 21 or 30 days after silking (DAS), and maturity for biomass and P uptake analysis in 2014-2015. Shoots of five consecutive plants were taken from each plot by cutting at the stem base, and separated into leaves, stem, and ears or kernels (when applicable). Additionally, developing ears from another three plants were harvested from silking to maturity approximately every 15 days in 2014 and at silking, 21 DAS, and maturity in 2015. Ear leaves at silking and 21 DAS (in 2015) or 30 DAS (in 2014) were sampled for determination of leaf total P and Pi concentrations.

In 2015-2016, P-deficient (P0) and P-sufficient (P150) plots were selected, and ear leaves and ears of three plants from each plot were harvested at silking and 21 DAS in 2015 (11:00 h and 21:00 h) and in 2016 (08:00 h and 17:00 h) for carbohydrate and anatomic analyses. Excised tissues were immediately stored in plastic bags in an ice box and transported to the laboratory for further processing. Ears were divided into cobs and florets (at silking) or kernels (at 21 DAS). In 2016, leaf disks (2 mm × 3 mm) for transmission electron microscopy (TEM) were collected from the longitudinally middle of the leaf blade and immediately fixed in 4% paraformaldehyde and 5% glutaraldehyde in 0.1 mol L^-1^ phosphate buffer (pH = 7.2).

### Dry Weight, C Concentration, and P Concentration

Whole-plant samples (leaves, stem, ears, or kernels) harvested in 2014-2015 were heat treated at 105°C for 30 min, dried at 65°C to a constant weight, weighed to obtain the DW, and ground to pass through a 1-mm sieve. An accurately weighed (around 0.4 g) ground materials were digested using a mixture of concentrated sulfuric acid and 30% H_2_O_2_, and P concentration determined using the molybdovanadate phosphate method (Soon and Kalra, 1995). To determine C concentration of kernels or florets, the samples were dried and ground, similarly to the whole-plant samples, but then further ground to a fine powder using a Retsch Ball Mill (Haan, Germany), and assayed with an Elementar vario Macro Cube CHNS analyzer (Langenselbold, Germany) with phenylalanine as a standard.

To determine total P and Pi in ear leaves, fresh tissues were taken from the whole leaf blade randomly (without the midrib) using 1 cm diameter puncher, and then divided into two groups. The first part (∼1 g) was homogenized using an IKA T10 tissue homogenizer (Wilmington, NC, United States) in 2% (v/v) acetic acid and extracted for 30 min, followed by centrifuging at 4000 g for 10 min at 4°C. The clear extracts were destined for Pi concentration determination using the phosphomolybdate method (Fredeen et al., 1989). The second part (∼2 g) was dried at 65°C, weighed, and digested as mentioned above for determination of total P concentration (Soon and Kalra, 1995). Inorganic P concentration was normalized using water content and expressed according to DW.

### Leaf Area, Green Leaf Longevity, and Photosynthesis

In 2014-2015, five uniform plants in each plot were marked. The maximum area of each leaf was calculated by the product of the measured leaf length × measured leaf width × 0.75, and green leaf longevity in 2014 (number of days from full expansion to 50% green leaf area of leaf) was recorded. Net photosynthesis (P_n_) was measured on the ear leaves at the middle part of the leaf blade from 09:00 h to 12:00 h at silking and at 15, 30, and 45 DAS. A portable photosynthesis system LI-COR LI-6400XT (Lincoln, NE, United States) was used with light supplied by a LI-6400-02B LED light source set at an irradiance of 1800 μmol m^-2^ s^-1^ (PAR), CO_2_ setting at 400 mmol m^-2^ s^-1^, flow rate at 500 mmol s^-1^, and the leaf temperature maintaining at 30 ± 1°C.

### Sugar and Starch Quantification

In 2015-2016, the harvested ear leaves and ears at silking and 21 DAS were lyophilized using a Zirbus VaCo 2 laboratory freeze dryer (Harz, Germany) and ground in a ball mill for 3 min with a frequency of 30 times per sec. Glucose, fructose and sucrose were extracted from the ground samples using a modified method of Zhao et al. (2010). Briefly, 50 mg dried tissues with 2.5 ml 80% (v/v) ethanol were heated in 80°C water bath for 10 min. Extracts were centrifuged and supernatants were collected. Each sample was extracted two more times and then supernatants were combined. Supernatants were heated in a 100°C water bath for 8 min with the reaction mixture of Fructose Assay Kit A085 or Sucrose Assay Kit A099-1 (Institute of Biological Engineering of Nanjing Jianchen, Nanjing, China). The changes in the absorbance of the hydrolysate were monitored to quantify fructose (at 285 nm) and sucrose (at 290 nm) against a blank with a Shimadzu UV mini-1240 spectrophotometer (Kyoto, Japan). For the glucose assay, supernatants were mixed with Glucose Assay Kit (CAT: 361500, Shanghai Rongsheng, Shanghai, China) and then measured in the spectrophotometer at 505 nm following the supplier’s protocol.

Starch concentration was determined using a kit for assay of total starch (K-TSTA 09/14, Megazyme International, County Wicklow, Ireland) following the supplier’s protocol. Briefly, 100 mg samples were pretreated with 80% ethanol to remove sugars and then hydrolyzed with thermal stable α-amylase and followed by amyloglucosidase. The product (glucose) was measured in the spectrophotometer at 510 nm and used to calculate starch concentration.

### Transmission Electron Microscopy (TEM) Observations

Tissues were fixed in 4% paraformaldehyde and 5% glutaraldehyde in 0.1 mol L^-1^ phosphate buffer (pH = 7.2). Subsequently, fixed tissues were rinsed with 0.1 mol L^-1^ phosphate buffer (pH = 7.2). Tissue samples were then stained with 1% osmium tetroxide in 0.1 mol L^-1^ phosphate buffer (pH = 7.2), followed by incubation at room temperature for 4 h, and then rinsed with the phosphate buffer. Samples were dehydrated in a graded ethanol series and transitioned into epoxy propane, then the dehydrated tissues were gradually infiltrated with Epon/Spurr’s resin and polymerized at 70°C for 12 h. Sections were cut using an Leica EM UC7 Ultramicrotome (Wetzlar, Germany) and a diamond knife, and post-stained using a lead citrate solution stain and uranyl acetate. Images were acquired with a JEOL JEM-1230 TEM (Tokyo, Japan). The starch granules and chloroplasts morphology were analyzed using ImageJ software (National Institutes of Health, Bethesda, Maryland, United States).

### Calculations and Statistical Analysis

The apparent P remobilization of whole-plant P from silking to *t* DAS (e.g., 30 DAS) was defined as the ratio of the difference in total P in vegetative parts (excluding grains) between silking and *t* DAS to total vegetative P at silking, based on Masoni et al. (2007). Apparent P remobilization from *t* DAS to maturity, as well as for leaf and stem tissues, was calculated analogous to the calculation for vegetative tissues from silking to *t* DAS.

Data were subjected to analysis of variance (ANOVA) using PROC ANOVA with the SAS package 9.1 (SAS Institute, Cary, NC). The least significant difference was used to compare the differences between treatment at a *P* < 0.05 level of probability. The relationships between C and P accumulation in developing ears were established using SigmaPlot (SigmaPlot10.0, United States).

## Results

### Influence of P Supply on Maize Growth, Photosynthesis, and Grain Yield

**FIGURE 1:**
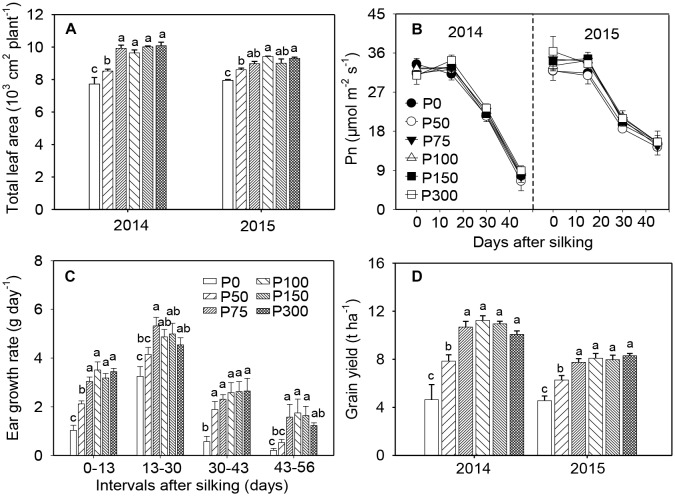
Total leaf area per plant **(A)**, photosynthesis in ear leaf **(B)**, ear growth rate in 2014 **(C)**, and grain yield **(D)** of maize supplied with low to high phosphorus inputs in 2014 and 2015. Bars represent the standard error of the mean (*n* = 4). Means with no letter in common are significantly different between treatments (LSD test, α = 5%).

In both years, P fertilization increased leaf growth and resulted in larger leaf area and longer longevity of green leaves, compared to the P0 treatment. No differences were observed among treatments receiving P75 (the corresponding Olsen-P concentration in 0–20 cm soil layer was 7.5 mg kg^-1^) and beyond (Figure 1A and Supplementary Figure S1). Photosynthesis of ear leaves was not influenced by soil-P supply at any examined stages during grain filling but began to decrease after 15 DAS (Figure 1B). During early ear development (from silking to 2–3 weeks after), the ear growth rate was greater in P50 treated plants than in the P0 plants, but both treatments exhibited slower growth rates than plants in P75. In all stages and P treatment combinations, no further increases in ear growth rates were observed when P supply increased from P75 to P300 (Figure 1C and Supplementary Figure S2). Consistent with the treatment effects on ear growth, grain yield across years in P75 was 0.52 to 1.30-fold higher than in P0 and 25–36% greater than in P50. No further increase in yield was obtained when P supply exceeded 75 kg ha^-1^ (Figure 1D). Similar trends were observed for shoot biomass as in grain yield (Supplementary Figure S3).

### Phosphorus Uptake, P-Use Efficiency, and P Redistribution

**FIGURE 2:**
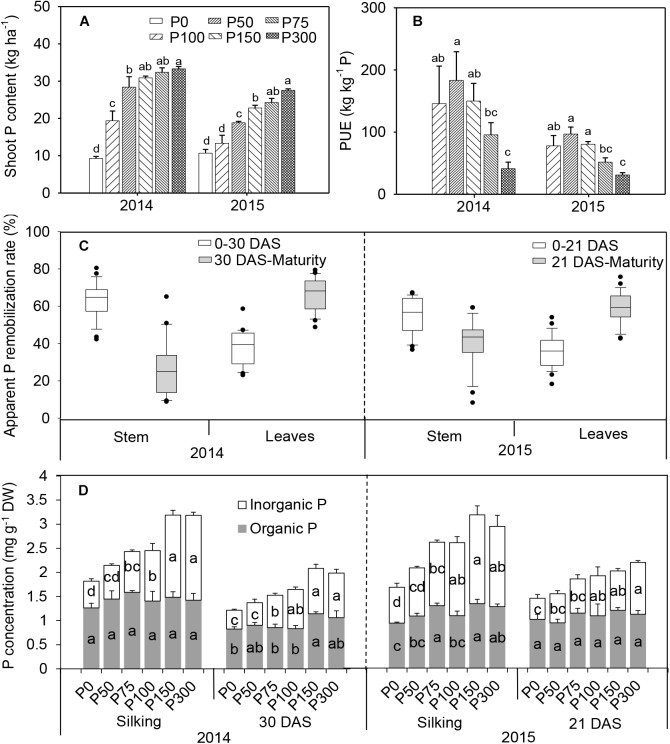
Total shoot P uptake **(A)**, P-use efficiency **(B)**, apparent P remobilization from stem and leaves **(C)**, and organic and inorganic P concentration in ear leaf **(D)** of maize supplied with low to high phosphorus inputs in 2014 and 2015. Bars represent the standard error of the mean (*n* = 4). Means with no letter in common are significantly different between treatments (LSD test, α = 5%). In panel **(C)**, the top and bottom edges of the boxes represent the 75 and 25 percentiles, and the top and bottom bars represent the 95 and 5 percentiles of all data, respectively.

Shoot-P uptake increased with the increase of P application, in contrast to dry matter accumulation, and the greatest value always occurred in the P300 treatment. A significant increase in P uptake occurred in P75 compared to P0 and P50 in 2015. Significantly more P uptake occurred was observed above P75 in 2014 and 2015, but there was no increase between P150 and P300 treatments (Figure 2A). Thus, increasing P fertilization decreased P-use efficiency i.e., yield produced per unit of fertilizer P (Figure 2B).

In all treatments, a remarkable P remobilization from vegetative organs (stem and leaves) was observed during grain filling (Figure 2C and Supplementary Figures S4, S5). For all treatments, apparent P remobilization from stems during the first half of the filling phase (silking to 30 DAS) in 2014 was 60–76%, which was more prominent than from leaves (30–44%). Evident P remobilization from leaves continued beyond 30 DAS, while apparent P remobilization from stems decreased dramatically or nearly stopped. A similar pattern was observed in 2015 (Figure 2C). Furthermore, similar patterns were found for the changes of P remobilization expressed in absolute terms (Supplementary Figure S4). Phosphorus starvation treatments (P0 and P50) caused a higher P remobilization (%) than other P-sufficiency treatments, especially in the older leaves in 2014 (Supplementary Figure S5).

Similar to the pattern of shoot P uptake, an increasing trend of total P concentration in ear leaves was observed at both silking and 21 DAS even with P inputs beyond 75 kg P_2_O_5_ ha^-1^ (Figure 2D). Increasing soil P availability led to greater Pi concentration in leaves (from 0.4–0.7 mg g^-1^ DW in P0 to 0.9–1.75 mg g^-1^ DW in P300), as well as higher proportion to total P (increased from 37 to 52% on average), while organic P concentrations were less responsive to P inputs (Figure 2D). When expressed on a leaf area basis, leaf Pi concentrations ranged from 0.46 to 0.87 mmol m^-2^ in P0 at silking and 30 DAS in 2014-2015, and even greater in the P fertilization treatments (from P50 to P300), mirroring partitioning patterns expressed on a DW basis in response to P availability (Supplementary Figure S6).

### Accumulation of Carbohydrates in the Ear Leaves in Response to P Availability

**FIGURE 3:**
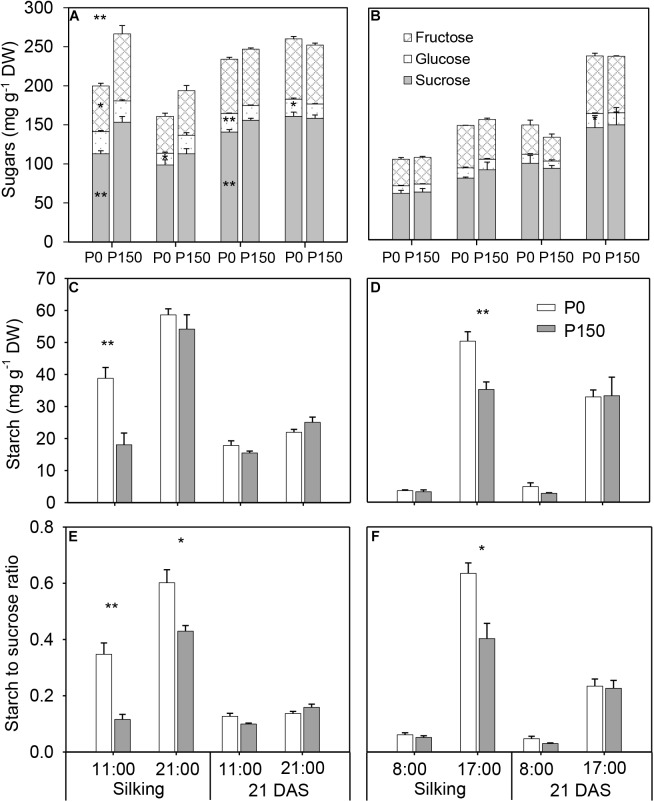
Concentrations of sugar **(A,B)** and starch **(C,D)**, and starch to sucrose ratio **(E,F)** in ear leaf of maize plants with low (P0) and high P (P150) supplies at silking and 21 DAS in 2015 **(A,C,E)** and 2016 **(B,D,F)**. Bars represent the standard error of the mean (*n* = 4). Statistical differences between P levels each year are marked with asterisks (^∗^*P* < 0.05, ^∗∗^*P* < 0.01).

Phosphorus starvation (P0) significantly reduced sucrose concentration at 11:00 h in the silking and 21 DAS sample time in 2015 in comparison with high P input (P150), as well as fructose, at silking. There were no significant differences in total soluble sugars (sucrose, glucose and fructose) between the P0 and P150 in other sample times, except at 11:00 h of silking in 2015 (Figure 3A,B). However, P-deficient leaves had similar or higher starch concentration compared to P-sufficient leaves, and the statistical increases were detected at silking (at 11:00 h in 2015 and 17:00 h in 2016). Pronounced diurnal patterns of starch turnover were observed at both silking and 21 DAS in 2 years, with greater accumulation at the end of day (Figure 3C,D). Overall, P deficiency led to starch to sucrose ratios in ear leaves that were similar to or higher than the ratios in ear leaves of high-P treatments, and the differences were statistically significant for the leaves at silking in both years, except at 08:00 h of silking in 2016 (Figure 3E,F).

**FIGURE 4:**
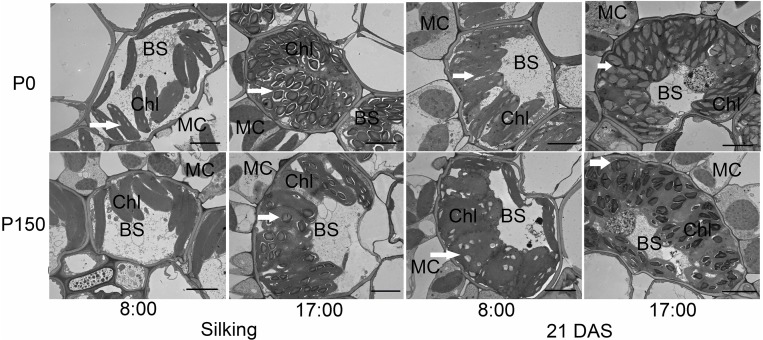
Transmission electron micrographs of bundle sheath cells with starch granules in ear leaves of phosphorus deficient (P0) and sufficient (P150) maize at 08:00 and 17:00 h of silking and 21 DAS in 2016. Arrows indicate the starch granules. BS, bundle sheath cell; Chl, chloroplast; and MC, mesophyll cell. Scale bars represent 5 μm.

**FIGURE 5:**
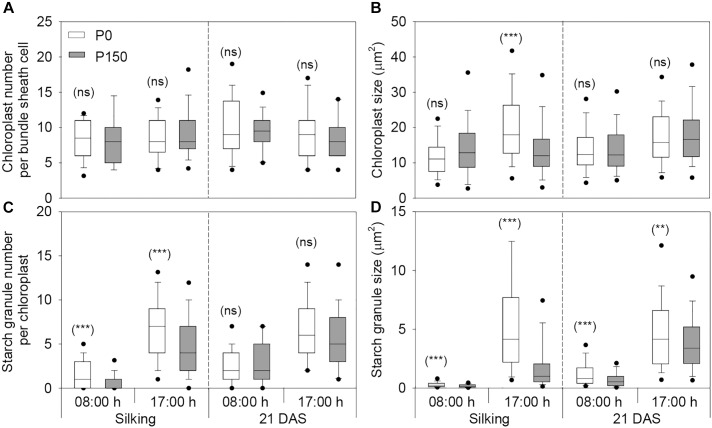
The number and size of chloroplasts **(A,B)** and starch granules **(C,D)** in bundle sheath cells of maize ear leaf at silking and 21 DAS in 2016. The solid lines within each box represent the median values of all data. The top and bottom edges of the boxes represent the 75 and 25 percentiles, and the top and bottom bars represent the 95 and 5 percentiles of all data, respectively. Statistical differences between P levels at each time point are marked with asterisks (^∗∗^*P* < 0.01, ^∗∗∗^*P* < 0.001; ns, not significant).

Phosphorus deficiency marginally affected the chloroplast number per bundle sheath cell and chloroplast size, except that larger chloroplasts were measured in P0 leaves sampled at 17:00 h of silking in 2016 (Figure 4, 5A,B). More starch granules were observed in ear leaves under P0 than P150 treatment at silking, while the differences were not significant at 21 DAS (Figure 4, 5C). The starch granules were consistently larger in P-deficient leaves than in P-sufficient leaves (*P* < 0.01). The granule size was increased by 0.8- to 1.8-fold at silking and 0.3- to 0.8-fold at 21 DAS, respectively (Figure 4, 5D).

### Sugar and Starch Concentrations in the Developing Ears as Affected by P Availability

**FIGURE 6:**
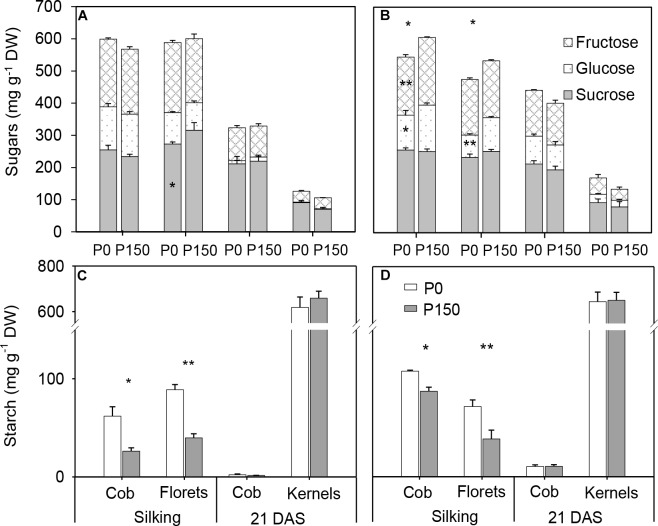
Sugar **(A,B)** and starch **(C,D)** concentration in the ears of phosphorus deficient (P0) and phosphorus sufficient (P150) maize plants at silking and 21 DAS in 2015 **(A,C)** and 2016 **(B,D)**. Bars represent the standard error of the mean (*n* = 4). Statistical differences between P levels in each year are marked with asterisks (^∗^*P* < 0.05, ^∗∗^*P* < 0.01).

At silking, P0 plants had similar concentrations of total soluble sugars in the cob and florets relative to P150 plants in 2015, while significant reductions were detected in the P-deficient plants in 2016 (Figure 6A,B). Interestingly, starch concentrations in P-deficient plants were greater than in P-sufficient plants in both the cob and florets (Figure 6C,D). From silking to 21 DAS, glucose, fructose and sucrose concentrations in the developing kernels dramatically decreased by 79–82% in P0 and P150 plants, which was coincident with a steep increase in starch concentration during the same time period (Figure 6). At 21 DAS, the sugar concentrations in the kernels tended to be slightly greater in P-deficient plants in comparison with P-sufficient plants, while the starch concentrations were similar. In addition, starch in the cob at this period was nearly depleted.

### Phosphorus and C Deposition in the Developing Grains

**FIGURE 7:**
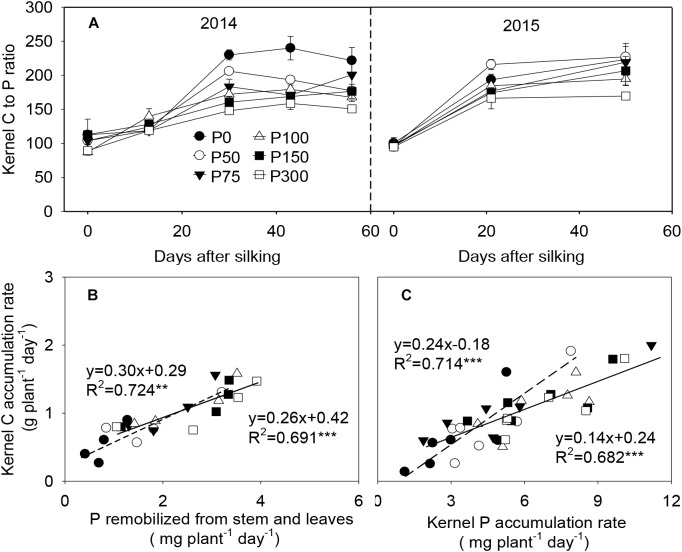
Carbon to phosphorus content ratio **(A)**, P remobilization rate from stem and leaves and carbon/phosphorus accumulation rate in the developing kernels of maize plants supplied with low to high phosphorus inputs **(B,C)** in 2014 and 2015. In panel **(A)**, bars represent the standard error of the mean (*n* = 4). In panels **(B,C)**, each data point is the mean of four replications. The short dash and solid lines represent regression under P deficiency (P0 and P50) and sufficiency (P75 to P300), respectively. ^∗∗^*P* < 0.01, ^∗∗∗^*P* < 0.001.

During grain filling, C to P ratios in the developing kernels increased from 87:1–113:1 at silking to 160:1–224:1 at 21 or 30 DAS, with greater values observed in the P0 and P50 plants. After 21 or 30 DAS, this ratio kept relatively constant in all treatments (Figure 7A). Kernel C and P accumulation rates were tightly and linearly correlated with each other, e.g., the C accumulation rate *vs*. the accumulation rate of P which was derived from stem and leaves remobilization (*r*^2^ = 0.69^∗∗∗^ to 0.72^∗∗^), and between C and total P accumulation rates in the developing kernels (*r*^2^ = 0.68^∗∗∗^ to 0.71^∗∗∗^). Interestingly, the correlation between kernel C *vs*. remobilized P was less affected by P availability, while the linear correlation between kernel C *vs*. total P accumulation was divided into two class types relating to low P (P0 and P50) and high P (P75 and beyond) (Figure 7B,C).

## Discussion

### Responses of Maize Growth and Grain Yield to High P Supply

Adequate application of P fertilizer is necessary for high crop yields, while excessive inputs to farmland are expensive and often lead to runoff losses of P and severe environmental issues, such as eutrophication (Pote et al., 1996; Aulakh et al., 2007; Bai et al., 2013). The annual P input in a maize-wheat cropping system in North China is more than 2-fold greater than crop removal, which far exceeds the levels of the United States and Northern European countries (Vitousek et al., 2009). Although modern maize hybrids exhibit greater P uptake capacity than older varieties (Ning et al., 2013), high P inputs beyond a threshold (∼75 kg P_2_O_5_ ha^-1^) failed to generate larger leaf area, faster ear growth, and more grain yields (Figure 1; Deng et al., 2014), which causes lower P-use efficiency. In this study, the Olsen-P concentration in the top 20 cm soil layer corresponding to P75 supply was 7.5 mg kg^-1^ (Supplementary Table S1), which is within the reported range of Olsen-P concentrations (7–15 mg kg^-1^) for optimal growth of maize grown under diverse environmental conditions and soil types (Mallarino and Atia, 2005; Colomb et al., 2007). Concomitantly, large amounts of P residue in the soil would exacerbate potential losses from soil erosion, runoff and leaching (Wang et al., 2011).

In contrast, shoot P uptake increased when P supply was over 75 kg ha^-1^, but did not contribute substantially to maize grain yield. Consistently, differences in P uptake by plants resulted in a large Pi concentration variability. Leaf Pi concentration exhibited an increased trend following increased P inputs, while organic P concentration in leaves was less responsive than the Pi fraction (Figure 2D and Supplementary Figure S6). Phosphorus in plants exists either as organic phosphate esters (nucleic acids, phospholipids, etc.) and phosphorylated proteins, or as free inorganic form (Pi) which can be further separated into two pools according to their physiological function, i.e., the metabolically active Pi in the cytoplasm and buffering Pi generally in the vacuoles (Veneklaas et al., 2012). The present results indicate that most of the excessive P uptake was distributed to the Pi fraction (Figure 2D), which was possibly stored within cell vacuoles, and acted as a P-buffering pool (Veneklaas et al., 2012). This may partially explain the stagnated increase in grain yield when P fertilization passed 75 kg P_2_O_5_ ha^-1^. Although the organic P pools were less responsive to P supply than Pi in leaves (Figure 2D and Supplementary Figure S6), the possibility cannot be excluded that each organic-P fraction shifts with P availability in maize leaves, following previous research reporting these changes in barley leaves in both absolute and in relative terms (Lambers et al., 2011). Further research is needed to know how organic P fractions respond to P availability and their impacts on C fixation and partitioning after silking in maize. Besides the well-documented concept of “soil critical P values” or “shoot P dilution curve” for maximum biomass production (Jones, 1983; Mallarino and Atia, 2005; Colomb et al., 2007), there could be a possible critical Pi concentration in leaves necessary for maximum grain yield, due to its sensitivity to plant-P status and soil-P supply (Bollons and Barraclough, 1999).

### Phosphorus Remobilization and Redistribution, and Impacts on Photosynthesis

Maintaining an adequate P concentration in vegetative organs, such as leaves, is essential for photosynthesis (Rychter and Rao, 2005). It has been revealed that leaf Pi concentration strongly correlates with rate of photosynthesis. The lowest Pi concentration for maximal photosynthesis was 0.6 mmol m^-2^ in leaves of seedling plants (Usuda and Shimogawara, 1991). In the present study, leaf Pi concentrations ranged from 0.46 to 0.87 mmol m^-2^ in P0 plants at silking and 30 DAS, and greater in other P fertilization treatments (Supplementary Figure S6). These values were close to or higher than the reported 0.6 mmol m^-2^ (Usuda and Shimogawara, 1991), which at least partially explained the very limited variation in photosynthetic rate between P treatments. For cereal crops, the obvious export of N and P from vegetative tissues generally occurs after flowering to support the new growth of developing seeds (Sklensky and Davies, 1993). There is a trade-off between the nutrient remobilization and photosynthesis maintenance in leaves during the reproductive period (Thomas and Ougham, 2014). The retention of P and N is beneficial for a longer leaf life-span and allows more C capture, while it may reduce the P-use efficiency, at least P remobilization efficiency (Veneklaas et al., 2012; Thomas and Ougham, 2014). Therefore, it is necessary to know how maize plants coordinate these two processes, i.e., P remobilization vs. C gain, and their responses to diverse P availability.

At the whole-plant level, the apparent P remobilization source during the first half of the grain filling phase was mainly the stem rather than the leaves (Figure 2C and Supplementary Figures S4, S5). This whole-plant P redistribution after silking is consistent with the pattern of N remobilization during the same development stage (Ning et al., 2017), implying that similar principles may be applied for post-silking redistribution of N and P. The priority of P remobilization from the stem indicated an effective strategy of P management, which to some extent preserves leaf photosynthetic capacity and allows for an extended period of C capture during grain filling. At the individual leaf level, the rates of photosynthesis in ear leaves were not affected by soil P supply at silking, or 15, 30, and 45 DAS, but decreased by 35–42% at 30 DAS relative to the values at silking across P treatments (Figure 1B). On the one hand, the results suggest that the decline in photosynthesis after silking is under genetic control and is less modified by P availability. Furthermore, maintenance of leaf photosynthesis in P-deficient plants was associated with a greater P investment to organic P pools including many photosynthetic metabolites. Ester P, which plays a role in photosynthetic and respiratory C metabolism, is generally lower than lipid P and nucleic acids, both in absolute and relative terms, under optimum P conditions. However, its proportion to total leaf P, such as in barley leaves, can be shifted from <5% under normal P supply up to 30% under severe P starvation (Lambers et al., 2011). These results imply that under severely P-impoverished conditions, plants invest more P to the photosynthetic and C metabolism, which highlights the crucial role of maintaining organic P levels for photosynthetic tissues (Veneklaas et al., 2012). During the first half of the grain filling phase, the organic P concentration in leaves under different P fertilization rates kept relatively stable, and the total leaf-P reduction was mainly attributed to Pi decline, which further protects organic P from losses (Figure 2D and Supplementary Figure S6). The results also imply that P remobilization from leaves preferentially occurs from Pi fractions, rather than organic P pools. Taken together, recycling of P for optimal plant C capture can be modulated by P management at the whole-plant level and/or by P redistribution within an individual leaf. Nonetheless, the total fixed C was less in P-deficient plants, corresponding to lower grain yield, due to the smaller leaf size and shorter leaf longevity.

### Source-Sink Carbohydrate Accumulation and Allocation in Response to P Supply

To further reveal the impacts of P availability on C production after silking, sugars and starch in both source leaves and developing ears were closely investigated under low (P0) and high (P150) P supply. Phosphorus starvation altered the pattern of carbohydrate partitioning in leaves, with less total soluble sugars and more starch accumulation at silking, as well as a higher starch to sucrose ratio (Figure 3). The results indicate that the fixed C by Calvin cycle had greater conservation within the leaf chloroplast rather than alternately being transported to the cytoplasm for sucrose biosynthesis under P deficiency. The syntheses of starch and sucrose are competing processes for substrate of triose phosphate. When the cytosolic Pi concentration is high, chloroplast triose phosphate is exported to the cytosol in exchange for Pi, and sucrose is synthesized (Taiz and Zeiger, 2010; Stitt and Zeeman, 2012). Otherwise, triose phosphate is retained within the chloroplast for starch synthesis when the cytosolic Pi concentration is low (Taiz and Zeiger, 2010; Stitt and Zeeman, 2012). Possibly, P starvation suppressed the levels of metabolically active Pi in leaf cytosol in the present study and built up more starch in the bundle sheath cells. In agreement with that, larger and more abundant starch granules were observed in the bundle sheath chloroplast under low P than high P supply, which, at least partially, explained starch accumulation in leaves under P-limiting conditions. It has been reported that in P-deficient soybeans, only 30% of the whole plant starch present at the end of a light phase was utilized during the subsequent 12-hour dark phase as compared with 68% for P-sufficient controls (Qiu and Israel, 1992). However, this was not the case in the present study, since the starch level in P-deficient leaves at the end of night was similar to that in P-sufficient plants in 2016 (Figure 3–5). In addition, the differences in starch accumulation between P supplies disappeared at the fast filling phase (21 DAS), which was likely due to the large demand for carbohydrates by developing kernels, when most of the carbohydrates in leaves are exported for biomass (Peng et al., 2014).

In developing ears, starch concentrations in both cob and florets of low-P plants were consistently greater than those of high-P plants, indicating that carbohydrates transported from leaves to ears were sufficient for the diminished sink under P-deficient conditions. Similar starch levels were observed at 21 DAS in the cob and developing kernels among P treatments, which implies that the pathway of starch biosynthesis was not inhibited in the kernels. The dramatic reduction of total soluble sugars in developing kernels from silking to 21 DAS under both low and high P, corresponding to a steep increase of starch concentration, further confirmed an active conversion from hexoses to starch synthesis (Bollons and Barraclough, 1999). It was speculated that yield losses of maize under P-limiting conditions were mainly attributed to lower sink size to accommodate the transported carbohydrates. In agreement with that, ear growth rate was drastically suppressed by P starvation (Figure 1C). It was stated that suppression of new growth in P-deficient plants is always likely to be larger than any effects on other processes, e.g., senescence (Veneklaas et al., 2012). Previous studies revealed that although shoot P concentration remained at relatively high levels, plant shoot growth would be repressed rapidly under P deficiency (Mimura et al., 1996; Lambers et al., 2008). Therefore, the new growth in the developing ears may be severely suppressed by P starvation, concomitantly causing a limited sink size to utilize the transported carbohydrates.

### Carbon and P Deposition in the Developing Ears

During the reproductive phase, a large amount of C and P into seeds occurs in annual grain crops (Sklensky and Davies, 1993; Veneklaas et al., 2012). Efficient use of P involves both C and P deposition in developing kernels, such as sufficient C export for biomass and high percentage remobilization from senescing leaves to young tissue. It is therefore important to understand how the fluxes of carbohydrates and P into grain are affected by P availability.

**FIGURE 8:**
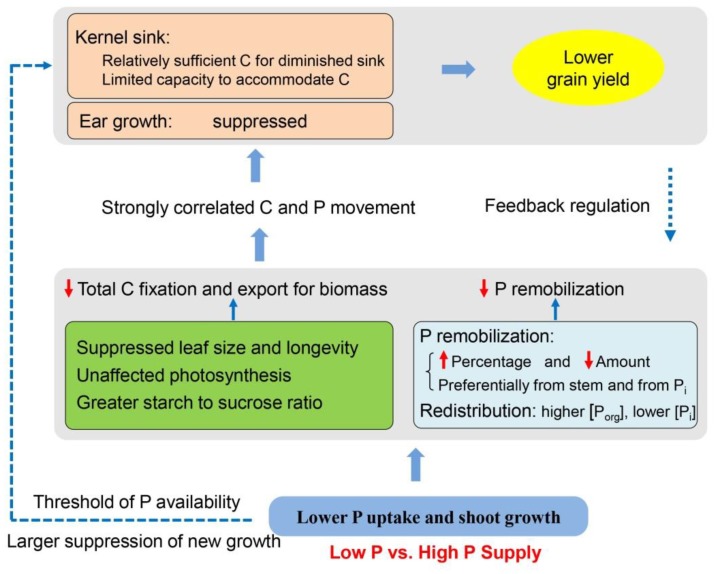
Schematic of a proposed model showing the source-sink carbon partitioning and phosphorus recycling relating to the grain yield in maize under low *vs*. high phosphorus supply. C, carbon; P, phosphorus; P_org_, organic P; P_i_, inorganic P.

Based on the results in this study and in the literature, we proposed an integrated framework of the P recycling and C partitioning in maize after silking and their effects on grain yield (Figure 8). Shoot growth and P uptake are repressed under P-limiting conditions (e.g., smaller leaf area and shorter leaf longevity), while photosynthetic rate is maintained due to the greater P investment to the organic pools than inorganic fractions, compared to shoot growth and P uptake in plants provided with a sufficient P supply. Regardless of P supply, results demonstrate that preferential P remobilization occurs from the stems than from leaves and has a priority for Pi fractions over organic P, further helping to protect the leaf function. P-starvation may result at a lower concentration of metabolically active Pi in the cytosol, leading to more triose-phosphate retention within the chloroplast for starch synthesis than export to cytosol for sucrose synthesis. Together with the smaller leaf size and lifespan, the total production of carbohydrates and export for growth are less in P-deficient plants than in P-sufficient plants. Phosphorus deficiency may also directly suppress the new growth of ears, generating limited sink size, which further limits both C and P movement to grains. Strong correlations were observed between C and P accumulation in the developing kernels, implying P movement to grain is likely driven by C allocation and demand within the plant (Marshall and Wardlaw, 1973; Peng and Li, 2005). The relationship between kernel C and remobilized P accumulation was less influenced by P availability. By contrast, the linear correlation between kernel C *vs*. total P accumulation was divided into two class types relating to low P and high P, suggesting a pivotal role of post-silking P uptake in this process under adequate P supply. However, the carbohydrates allocated to the developing ear seems adequate to match the diminished sink capacity in P-deficient plants, possibly because of the reduced sink size to constrain carbohydrate accommodation and the grain yield under P starvation. In turn, the diminished sink capacity imposes a feedback regulation reducing the C and P transport from source tissues. Increasing P fertilizer supply benefits shoot growth and P uptake, but the grain yields stagnate when P supply passes a threshold (∼75 kg P ha^-1^), which lowers P-use efficiency.

## Data Availability

All datasets generated for this study are included in the manuscript and/or the Supplementary Files.

## Author Contributions

CW and PN conceived and designed the field experiment, and analyzed the data. CW performed the experiments. PN and CW wrote the manuscript.

## Conflict of Interest Statement

The authors declare that the research was conducted in the absence of any commercial or financial relationships that could be construed as a potential conflict of interest.
