# Supplementary material for: Post-silking Phosphorus Recycling and Carbon Partitioning in Maize Under Low to High Phosphorus Inputs and Their Effects on Grain Yield
Source: Front Plant Sci. 2019 Jun 12;10:784. doi: 10.3389/fpls.2019.00784 (PMC6582669; doi:10.3389/fpls.2019.00784)
Supplement: Supplementary file 1 [file Table_1.DOCX]

**Supplementary data**

**Table S1** Basic soil properties in the topsoil (0-20 cm) and subsoil (20-40 cm) of plots receiving different P fertilizer input before sowing.

| Soil layer (cm) | | P0 | P50 | P75 | P100 | P150 | P300 |
| --- | --- | --- | --- | --- | --- | --- | --- |
| CaCl_2_ extractable mineral N | 0-20 | 13.4 | 13.6 | 11.9 | 12.1 | 13 | 12.1 |
| （mg kg^-1^） | 20-40 | 8.7 | 8.9 | 9.1 | 8.8 | 7.8 | 7.9 |
| NH_4_OAc extracted K | 0-20 | 107.6 | 117.6 | 114.5 | 110.8 | 114.5 | 112.5 |
| （mg kg^-1^） | 20-40 | 97.6 | 89 | 84 | 85 | 87 | 90.9 |
| organic matter (g kg^-1^) | 0-20 | 12.1 | 12.3 | 11.9 | 11.7 | 12.0 | 11.8 |
|  | 20-40 | 9.4 | 8.0 | 8.5 | 9.3 | 8.7 | 8.8 |
| pH | 0-20 | 8.6 | 8.2 | 8.3 | 8.4 | 8.3 | 8.1 |
|  | 20-40 | 8.3 | 8.2 | 8.2 | 8.3 | 8.1 | 8.3 |
| Olsen-P（mg kg^-1^） | 0-20 | 3.6 | 5.8 | 7.5 | 10.3 | 16.1 | 20.1 |
|  | 20-40 | 3.2 | 4.3 | 4.8 | 5.9 | 6.4 | 7.8 |
| CEC (cmol kg^-1^) | 0-20 | 15.4 | 15.6 | 16.3 | 16.3 | 15.8 | 14.5 |
|  | 20-40 | 15.2 | 15.8 | 16.9 | 16.7 | 16.7 | 15.6 |

CEC Cation Exchange Capacity

**Fig. S1** The maximum green leaf area at silking and green leaf longevity of maize supplied with different phosphorus rates (kg P_2_O_5_ ha^-1^) in 2014.

**Fig. S2** Ear growth rate of maize plants supplied with different phosphorus rates in kg ha^-1^ in 2015. Bars represent the standard error of the mean (*n* = 4). Bars with no letter in common represent significant differences between treatment (LSD test, α = 0.05).

**Fig. S3** Shoot biomass at maturity of maize plants supplied with different phosphorus rates (kg ha^-1^) in 2014-2015. Bars represent the standard error of the mean (*n* = 4). Bars with no letter in common represent significant differences between treatment (LSD test, α = 0.05).

**Fig. S4** Apparent phosphorus remobilization (quantity) from leaves and stem of maize plants supplied with different phosphorus rates (kg ha^-1^ P0-P300) during grain filling in 2014 and 2015. DAS, days after silking. Each data point is the mean of four replications with bars representing the standard error of the mean (*n* = 4).


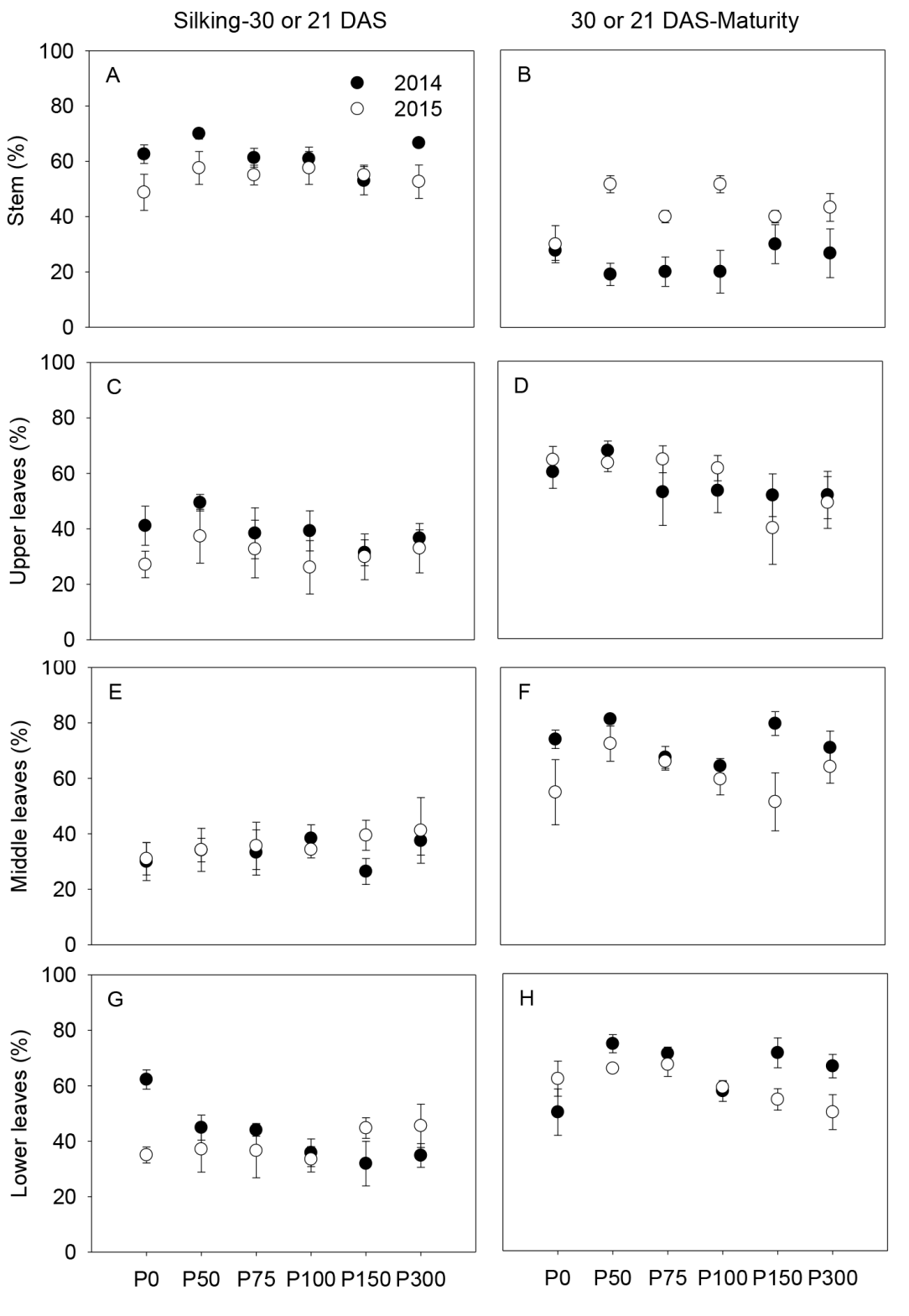


**Fig. S5** Apparent phosphorus remobilization (%) from lower, middle, upper leaves and stem of maize plants supplied with different phosphorus rates (0-300 kg ha^-1^) during grain filling in 2014 and 2015. Middle leaves, three leaves including ear leaf, one leaf above and one leaf below ear leaf; upper leaves, all the leaves above the middle leaves; lower leaves, all the leaves below the middle leaves. DAS, days after silking. Each data point is the mean of four replications with bars representing the standard error of the mean (*n* = 4).


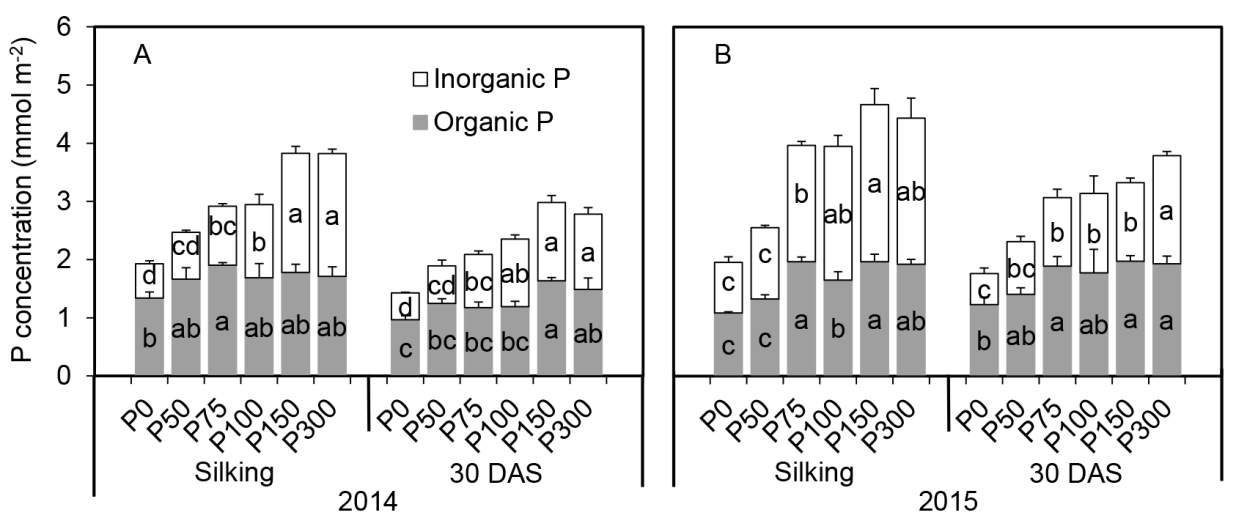


**Fig. S6** Organic and inorganic P concentration based on leaf area in ear leaf of maize supplied with low to high phosphorus inputs in 2014 and 2015. Bars represent the standard error of the mean (*n* = 4). Means with no letter in common are significantly different between treatments (LSD test, α = 5%).
